# Supplementary material for: Design and Testing of Mobile Laboratory for Mitigation of Gaseous Emissions from Livestock Agriculture with Photocatalysis
Source: Int J Environ Res Public Health. 2021 Feb 5;18(4):1523. doi: 10.3390/ijerph18041523 (PMC7915192; doi:10.3390/ijerph18041523)
Supplement: Supplementary file 1 [file ijerph-18-01523-s001.pdf]

International Journal of
Environmental Research and Public Health

MDPI

Supplementary Materials

Design and testing of mobile laboratory for mitigation of gaseous emissions from livestock agriculture with photocatalysis

Myeongseong Lee [1], Jacek A. Koziel [1,*], Wyatt Murphy [1], William S. Jenks [2], Blake Fonken [1], Ryan Storjohann [1], Baitong Chen [1], Peiyang Li [1], Chumki Banik [1], Landon Wahe [1], Heekwon Ahn [3]

[1] Department of Agricultural and Biosystems Engineering, Iowa State University, Ames, IA 50011, USA; leefame@iastate.edu (M.L.), koziel@iastate.edu (J.K.), wmurphy@iastate.edu (W.M.), bjfonken@iastate.edu (B.F.), rls1@iastate.edu (R.S.), baitongc@iastate.edu (B.C.), peiyangl@iastate.edu (P.L.), cbanik@iastate.edu (C.B.), lwahe@iastate.edu (L.W.)

[2] Department of Chemistry, Iowa State University, Ames, IA 50011, USA; wsjenks@iastate.edu (W.J.)

[3] Department of Animal Biosystems Sciences, Chungnam National University, Daejeon 34134, Korea; hkahn@cnu.ac.kr (H.A.)

* Correspondence: koziel@iastate.edu; Tel.: +1-515-294-4206

Supplementary Material 1. Experimental mobile laboratory setup

The mobile laboratory (7.2 × 2.4 × 2.4 m) was designed to evaluate the effectiveness of UV photocatalysis. The treatment chambers (7.2 × 0.9 × 2.4 m) were partitioned out of the mobile lab interior (Figure S.1). Vertical baffles constructed of pine wood board (5 × 10 cm; 2 × 4 in) (Lowes, Mooresville, NC, USA) with embossed white fiberglass reinforced plastic (FRP) wall panels (3 mm; 1/8 in thickness, Lowes, Mooresville, NC, USA). The mobile laboratory consists of a series of 12 chambers. The last two chambers of the 12 chambers (#11 and #12, closest to the outlet) are constructed as one double size chamber with no vertical baffles in the middle to allow ample room for volumetric flow measurement. Untreated air is brought in through the inlet (right, red) and treated while flowing in a serpentine pattern from the inlet (right, red) to the outlet (left, blue). There are two fans (I-Fan Type 40, 18 in, Variable Speed, Fancom, Panningen, The Netherlands) at the front (chamber #1) and the last chamber (chamber #11-12) in the mobile lab. The fan in chamber #1 is located parallel to the vertical baffle, and the fan in the chamber #11-12 is perpendicular to the vertical baffles. The fans installed in chamber #1 and chamber #11-12 are the same size fan (0.5 × 0.5 × 0.1 m) with an opening diameter of 0.5 m (18 in) and have the same maximum velocity. The fan in chamber #1 draws in untreated air, and the fan in chamber #11-12 expels treated air out. These two fans control the airflow rate by setting the percentage of the maximum volumetric flow rate of the fan using a Lumina controller (Lumina 20/21, Fancom, Panningen, The Netherlands). These two fan's volumetric flow rates are controlled separately to allow positive-pressure ventilation and negative-pressure ventilation of the mobile laboratory chambers. An anemometer fan (ATM, Fancom, Panningen, The Netherlands) in the 10th chamber exists to measure the volumetric flow rate created by the two fans. Chambers #11 and #12 are combined to allow unblocked airflow through the ATM unit, as mentioned above. The measured ventilation is expressed as the percentage of the maximum volumetric flow rate of the fans through the Lumina controller.

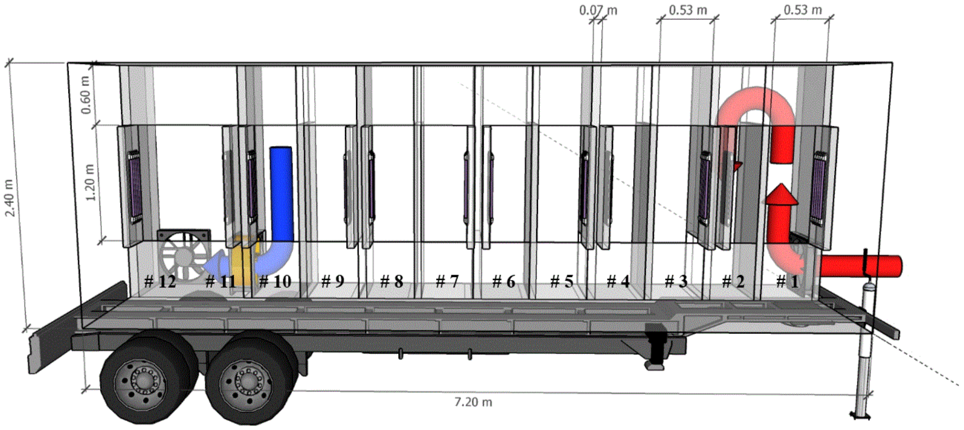

Figure S.1. Schematic of a flow-through reactor for UV treatment of gaseous emissions (side view of mobile laboratory). The mobile laboratory consists of a series of chambers (#1-#12) equipped with photocatalytic surfaces and UV lamps. Treated air is moving in a serpentine pattern from the inlet (right, red) to the outlet (left, blue). UV lamps are mounted on doors to chambers (doors are closed during UV treatment). The anemometer fan (yellow) continuously measures the volumetric flow rate through the mobile lab.

Five LED UV-A lamps (T8 LED, Eildon Technology, Shenzhen, China) were mounted on doors to each chamber (#1-#12, The doors are closed during UV treatment, Figure S.2). Rubber seals were used between the door and the chamber to prevent leakage. A total of 11 panels with TiO2 coating (nanostructured TiO_2 anatase at 10 µg·cm^{-2} from PureTi, Cincinnati, OH, USA) was equipped in each chamber (Figure A2). The 11 panels (5.1 m^2) accounted for about 76% of the surface area of one chamber (6.7 m^2).

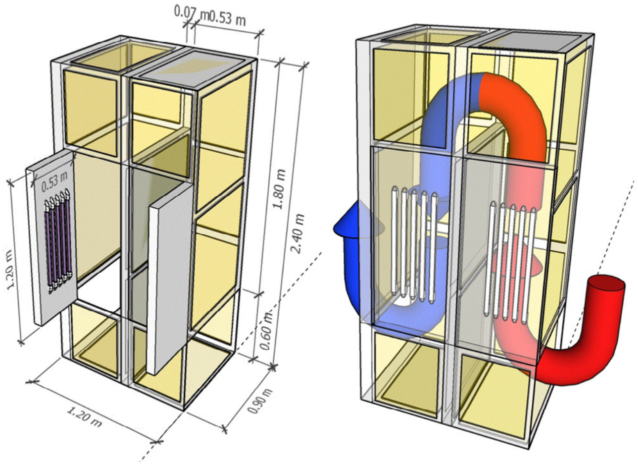

(a) (b)

Figure S.2. Schematic of UV treatment chambers with TiO_2 coated panels (yellow). A total of eleven panels coated with TiO_2 are attached in one chamber. (a): dimensions; (b): airflow, red: untreated air irradiated with UV light, blue: treated air;

Supplementary Material 2. UV irradiation source installed in the mobile laboratory

A total of 60 UV-A LED lamps (T8 LED, Eildon Technology, Shenzhen, China) were installed in 12 chambers. LED UV-A lamp was used as a commercially available product in the market. The output of these lamps at a 1 m distance between it and a sensor in the laboratory is shown in Table S.1.

Table S.1. Experimental UV-A lamp specification

	**LED**
Total light intensity ($mW \cdot cm^{-2}$)	0.03
Total electric power consumption (W)	15.8
Luminous efficacy (provided by the lamp manufacturer)	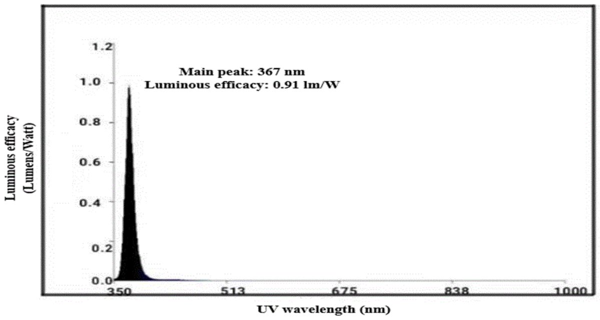

In the UV treatment chamber, the light intensity of photolysis and photocatalysis were investigated for their effects on treated gases when UV-A light irradiated (Figure S.3). Photolysis light intensity refers to the light intensity directly irradiated to the gas from the installed lamp. Photocatalysis light intensity means the light intensity irradiated on the panel surface installed in the UV chamber. Photolysis light intensity was measured in six directions from three points (Bottom: 0.6 m from the floor, Mid: 1.2 m, Top: 1.8 m) in the chamber. Using a box made of styrofoam that fits tightly to the chamber size (0.3 × 0.5 × 0.2 m), a light intensity sensor was installed in the three points at the center of the chamber (Figure A.21), and the sensor was rotated in six directions to measure. The light intensity of photocatalysis was measured in each of the 11 installed panels (Top, Bottom, Front Top, Front Bottom, Left Top, Left Bottom, Right Top, Right Bottom, Back Top, Back Middle, and Back Bottom). The light intensity was measured at 0.2 x 0.2 m intervals on the surface of the panels installed.

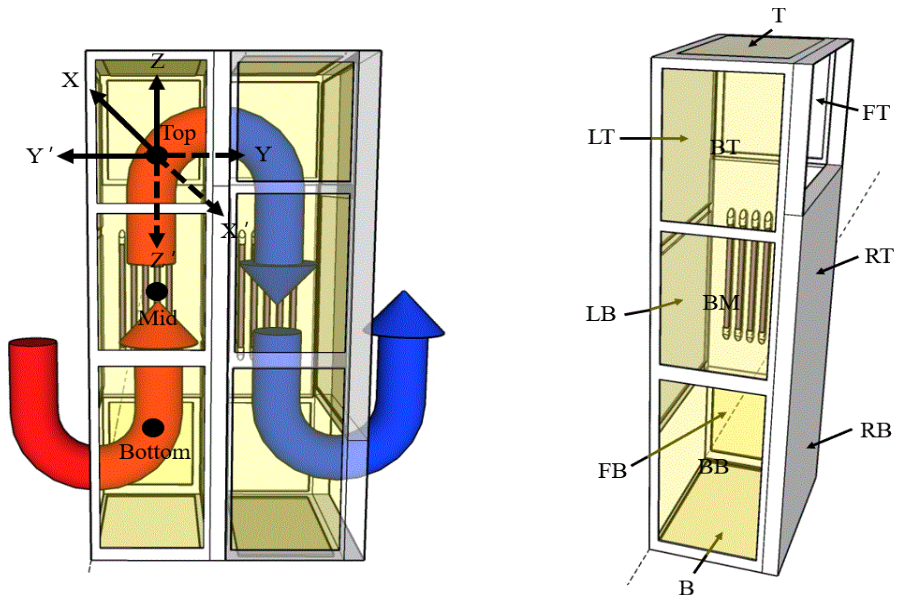

(a) (b)

Figure S.3. Measurement of light intensity in the UV treatment chamber. (a): the light intensity of photolysis, the light intensity irradiated directly from UV sources were investigated at three points (Top, Mid, and Bottom) in the chamber, and the light intensity was measured in six directions (X: front, X': back, Y: right, Y': left, Z: top, Z': bottom) at each point to obtain the average and total value; (b): the light intensity of photocatalysis, The light intensity irradiated was measured to a total of eleven coated panels (T, B, F.T., F.B., L.T., L.B., R.T., R.B., B.T., B.M., and B.B.) with TiO_2 in one chamber;

In addition, a portable UV lamp holder was installed to provide more light intensity in the chamber (Figure S.4). The portable lamp holder was made in two sizes (small: 0.5 x 1.7 m, large: 0.7 x 1.7 m). The small size holder can be installed with 5 lamps on the front and back for a total of up to 10 lamps. Also, it was made for installation on the backside of the chamber. The large size holder can be installed with 10 lamps on the front and the back, respectively. It was made for installation on the side of the chamber. A total of 55 lamps were installed in two chambers (#2 and #3) with portable UV lamp holders (including the 5 lamps installed on the door). Therefore, a total of 160 UV-A lamps was installed in the 12 chambers of the mobile laboratory.

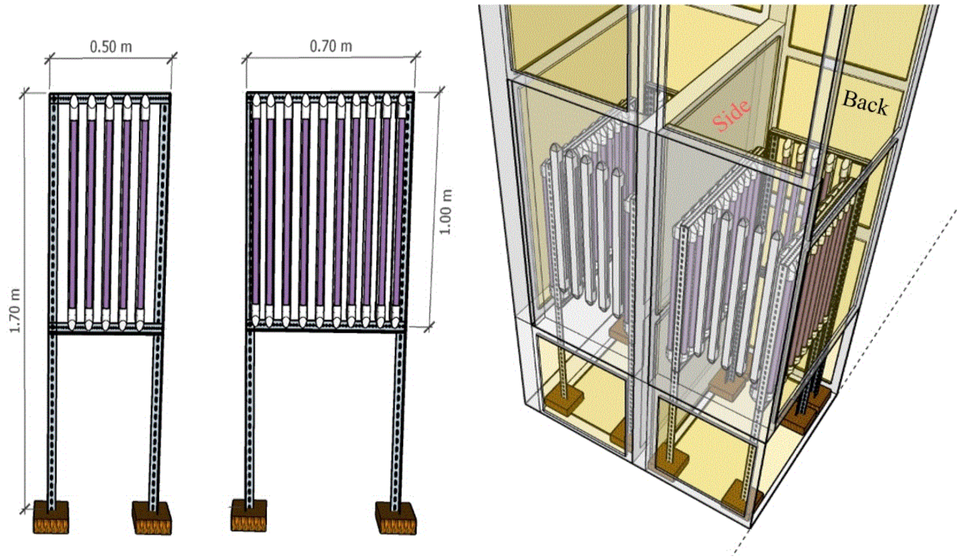

(a) (b)

Figure S.4. Portable UV lamp holder for increasing light intensity inside selected two chambers (#2 and #3). A total of 10 lamps (20 lamps) can be installed on both sides of the short-length (long-length, 0.7 m) portable UV lamp holder. (a): dimensions; (b): schematic of portable UV lamp holder inside two chambers; Up to 55 lamps were installed in one chamber to investigate the reduction of the target gas according to the increase in light intensity, 20 lamps were arranged on the side, and 10 lamps were arranged on the back;

Supplementary Material 3. The results of UV light intensity

The LED UV-A lamp showed a stable light intensity immediately after turning it on, unlike a fluorescent lamp (Figure S.5). Therefore, it was investigated that treatment time is not required for the stable light intensity of the LED lamp.

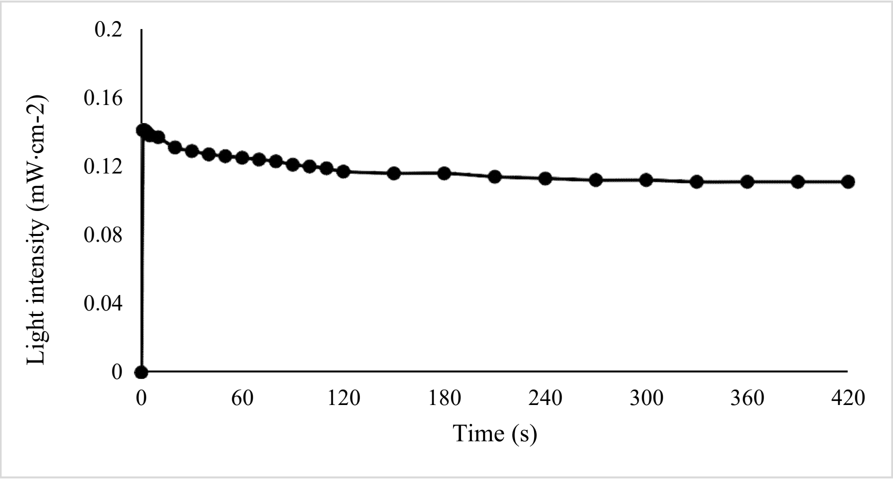

Figure S.5. The measured UV light intensity from a cold start lamp. LED lamps to exhibit relatively fast performance, i.e., no apparent delay in full light intensity. Light intensity was obtained by irradiating light from 5 lamps at a distance of 1m.

The photolysis light intensity measured in the chamber of the mobile lab showed in Table A4-A8. The average light intensity in 6 directions measured at 3 points was shown in Table S.2. The light intensity measured in 6 directions showed considerable variation. For example, 0.3 mW·cm^{-2} was detected in the forward direction that was directly illuminated. However, the ~ 100x low light intensity was observed in the direction indirectly illuminated. However, the average and total sum of light intensity were similar in all chambers (Figure S.6). As the number of lamps installed in the chamber increased, the light intensity was also increased.

Table S.2. The measured light intensity (I, mW·cm^{-2}) of photolysis with increasing LED UV-A lamps in chamber #2.

Lamp quantity	X (front)	Y (right)	Z (top)	X' (back)	Y' (left)	Z' (bottom)	Average (Average of X,X',Y,Y',Z,Z')	Sum (X+X' + Y+Y' +Z+Z')
5	0.34	$0.24\cdot10^{-2}$	$0.43\cdot10^{-2}$	$0.36\cdot10^{-2}$	$0.25\cdot10^{-2}$	$0.24\cdot10^{-2}$	0.06	0.36
20	0.46	0.81	$0.43\cdot10^{-1}$	0.22	0.77	$0.74\cdot10^{-1}$	0.40	2.38
30	0.52	0.93	0.11	0.68	0.91	0.37	0.59	3.52
40	0.66	1.1	0.14	0.67	1.02	0.59	0.70	4.18
55	0.68	1.3	0.17	0.84	1.4	0.87	0.88	5.26

Note: X, X' Y, Y", Z, and Z' refer to the six spatial coordinates inside a chamber illustrated in Figure 3;

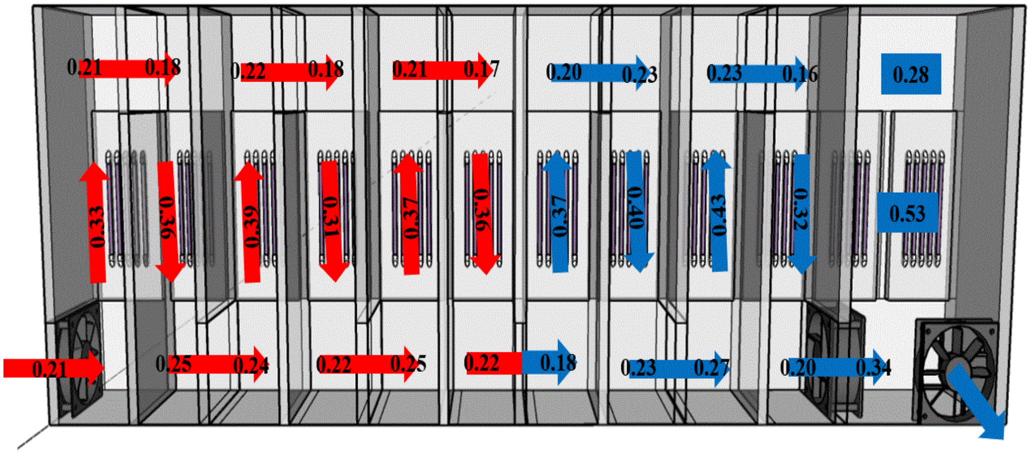

Figure S.6. The measured light intensity of photolysis (mW·cm^{-2}), The sum of the light intensity radiated on the treated gas from six directions. Treated air is moving from chamber #1 (inlet; left) to chamber #11&12 (right; outlet). Chamber #1 to #10 are geometrically identical, while chamber #11&12 is double-wide, I.e., does not have a partition in the middle.

The light intensity map of photocatalysis irradiated on the TiO_2 coated panels is presented as Figure S.7-S.11. The photocatalysis light intensity also showed a large difference gap about 1000 times between the directly irradiated part and the non-directed part (Table S.3). However, there was no area in the panel where no light intensity was detected. The result of the increased light intensity

using a portable UV lamp holder was shown in Table S.3. As the number of lamps installed in the chamber increased, the light intensity was also increased. The measured light intensity of photolysis and photocatalysis were summarized in Table S.2-8 and Figure S.7-11.

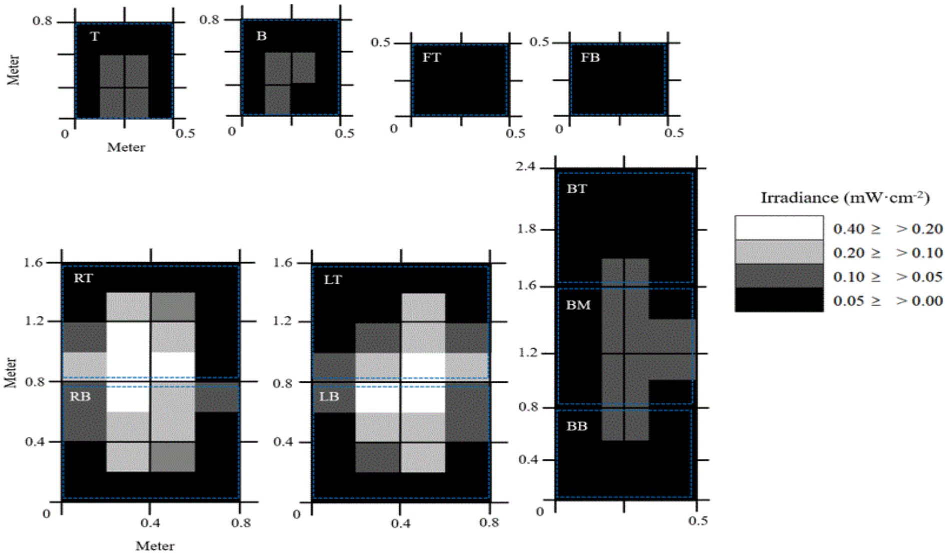

(a)

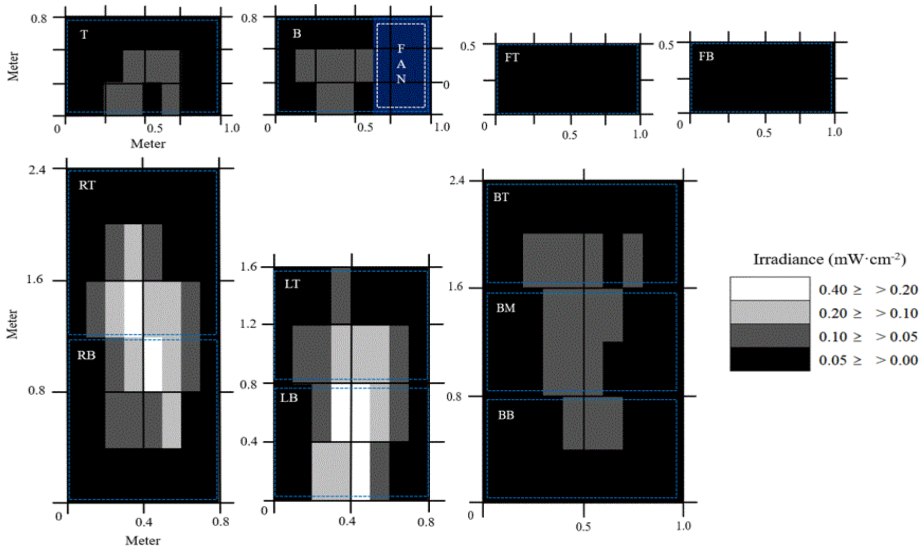

(b)

Figure S.7. The measured light intensity of photocatalysis. Map of UV-A light intensity measured on the surface of eleven panels inside a single chamber illustrated in Figure 3. (a) Light intensity irradiated to the panel surface from chambers #2 ; (b) Light intensity irradiated to the panel surfaces of double-wide chamber #11 and #12;

Table S.3. UV-A light intensity (I, mW·cm^{-2}) of photocatalysis at 11 panels in #2 chamber (Top, Bottom, Front Top, Front Bottom, Left Top, Left Bottom, Right Top, Right Bottom, Back Top, Back Middle, and Back Bottom; location of panels was illustrated in Figure 3).

Quantity of lamps	T	B	FT	FB	RT	RB	LT	LB	BT	BM	BB	Average (of 11 panels)
5	0.02	0.02	$0.02\cdot10^{-3}$	$0.02\cdot10^{-3}$	0.10	0.08	0.08	0.09	0.01	0.04	0.01	0.05±0.08
20	0.03	0.03	$0.02\cdot10^{-1}$	$0.01\cdot10^{-1}$	0.14	0.13	0.11	0.14	0.02	0.10	0.03	0.08±0.13
30	0.06	0.05	0.03	0.02	0.27	0.26	0.21	0.25	0.06	0.30	0.03	0.17±0.26
40	0.10	0.08	0.06	0.03	0.58	0.54	0.45	0.55	0.08	0.37	0.03	0.32±0.38
55	0.16	0.12	0.08	0.06	0.83	0.79	0.61	0.73	0.26	0.76	0.13	0.49±0.53

Supplementary Material 4. Summary of photolysis light intensity

Table S.4. Measured light intensity (367 nm, mW·cm^{-2}) measured at 6 directions in each chamber (front, back, right, left, top, bottom directions were illustrated in Figure 3) in three locations (top, middle, bottom). **Five** UV-A lamps were turned 'on' inside chamber #2 during measurement.

Location	X	Y	Z	X'	Y'	Z'	Average (of X,X',Y,Y',Z,Z')
Top	0.18	$0.07\cdot10^{-2}$	$0.74\cdot10^{-3}$	$0.87\cdot10^{-3}$	$0.24\cdot10^{-3}$	$0.86\cdot10^{-3}$	$0.31\cdot10^{-1}$
Middle	0.34	$0.24\cdot10^{-2}$	$0.43\cdot10^{-2}$	$0.36\cdot10^{-2}$	$0.25\cdot10^{-2}$	$0.24\cdot10^{-2}$	$0.59\cdot10^{-1}$
Bottom	0.24	$0.14\cdot10^{-2}$	$0.34\cdot10^{-3}$	$0.18\cdot10^{-2}$	$0.91\cdot10^{-3}$	$0.17\cdot10^{-3}$	$0.41\cdot10^{-1}$

Table S.5. Measured light intensity (367 nm, mW·cm^{-2}) measured at 6 directions in each chamber (front, back, right, left, top, bottom directions were illustrated in Figure 3) in three locations (top, middle, bottom). **Twenty** UV-A lamps were turned 'on' inside chamber #2 during measurement.

Location	X	Y	Z	X'	Y'	Z'	Average (of X,X',Y,Y',Z,Z')
Top	0.20	$0.37\cdot10^{-2}$	$0.71\cdot10^{-3}$	$0.13\cdot10^{-2}$	$0.34\cdot10^{-2}$	$0.19\cdot10^{-2}$	$0.35\cdot10^{-1}$
Middle	0.46	0.81	$0.43\cdot10^{-1}$	0.22	0.77	$0.74\cdot10^{-1}$	0.40
Bottom	0.31	$0.96\cdot10^{-2}$	$0.35\cdot10^{-2}$	$0.19\cdot10^{-2}$	$0.10\cdot10^{-1}$	$0.51\cdot10^{-3}$	$0.56\cdot10^{-1}$

Table S.6. Measured light intensity (367 nm, mW·cm^{-2}) measured at 6 directions in each chamber (front, back, right, left, top, bottom directions were illustrated in Figure 3) in three locations (top, middle, bottom). **Thirty** UV-A lamps were turned 'on' inside chamber #2 during measurement.

Location	X	Y	Z	X'	Y'	Z'	Average (of X,X',Y,Y',Z,Z')
Top	0.37	$0.24\cdot10^{-1}$	$0.12\cdot10^{-2}$	$0.27\cdot10^{-2}$	$0.11\cdot10^{-1}$	0.16	$0.95\cdot10^{-1}$
Middle	0.52	0.93	0.11	0.68	0.91	0.37	0.59
Bottom	0.43	$0.41\cdot10^{-1}$	0.22	$0.41\cdot10^{-2}$	$0.19\cdot10^{-1}$	$0.17\cdot10^{-2}$	0.12

Table S.7. Measured light intensity (367 nm, $mW \cdot cm^{-2}$) measured at 6 directions in each chamber (front, back, right, left, top, bottom directions were illustrated in Figure 3) in three locations (top, middle, bottom). **Forty** UV-A lamps were turned 'on' inside chamber #2 during measurement.

Location	X	Y	Z	X'	Y'	Z'	Average (of X,X',Y,Y',Z,Z')
Top	0.51	0.16	$0.24 \cdot 10^{-2}$	$0.72 \cdot 10^{-2}$	0.07	0.41	0.19
Middle	0.66	1.10	0.14	0.67	1.02	0.59	0.70
Bottom	0.59	0.39	0.47	$0.84 \cdot 10^{-2}$	0.19	$0.22 \cdot 10^{-2}$	0.28

Table S.8. Measured light intensity (367 nm, $mW \cdot cm^{-2}$) measured at 6 directions in each chamber (front, back, right, left, top, bottom directions were illustrated in Figure 3) in three locations (top, middle, bottom). **Fifty-five** UV-A lamps were turned 'on' inside chamber #2 during measurement.

Location	X	Y	Z	X'	Y'	Z'	Average (of X,X',Y,Y',Z,Z')
Top	0.55	0.32	$0.12 \cdot 10^{-1}$	$0.31 \cdot 10^{-1}$	0.19	1.02	0.35
Middle	0.68	1.30	0.17	0.84	1.40	0.87	0.88
Bottom	0.61	0.58	0.84	$0.22 \cdot 10^{-1}$	0.48	$0.31 \cdot 10^{-1}$	0.43

Supplementary Material 5. Summary of photocatalysis light intensity

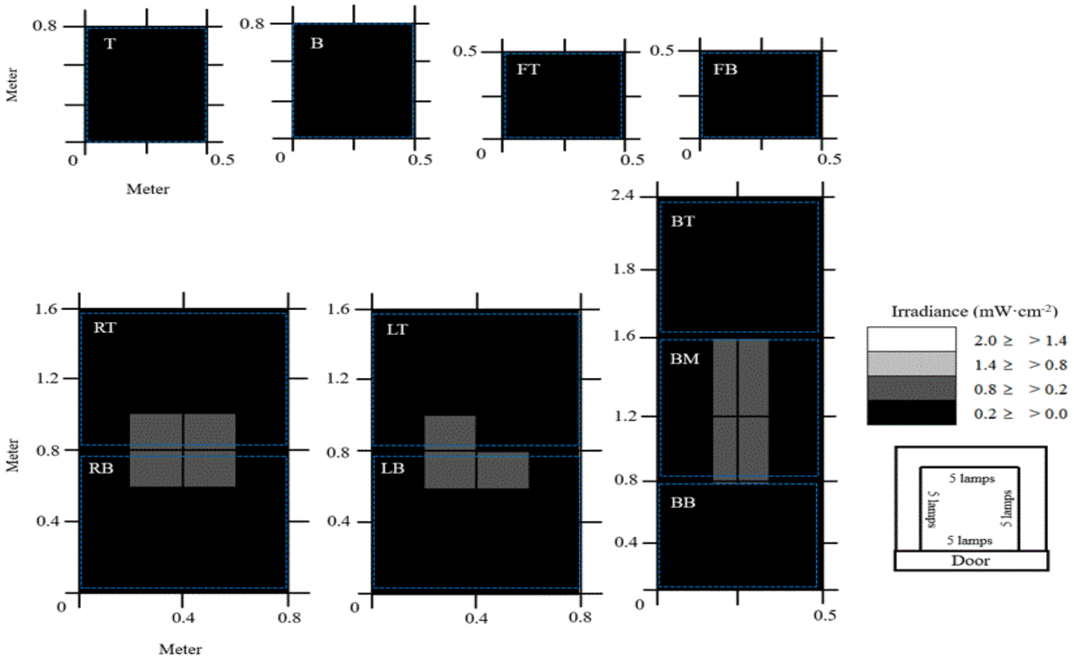

Figure S.8. The measured light intensity of photocatalysis. Map of UV-A light intensity measured on the surface of eleven panels inside a single chamber with 20 lamps.

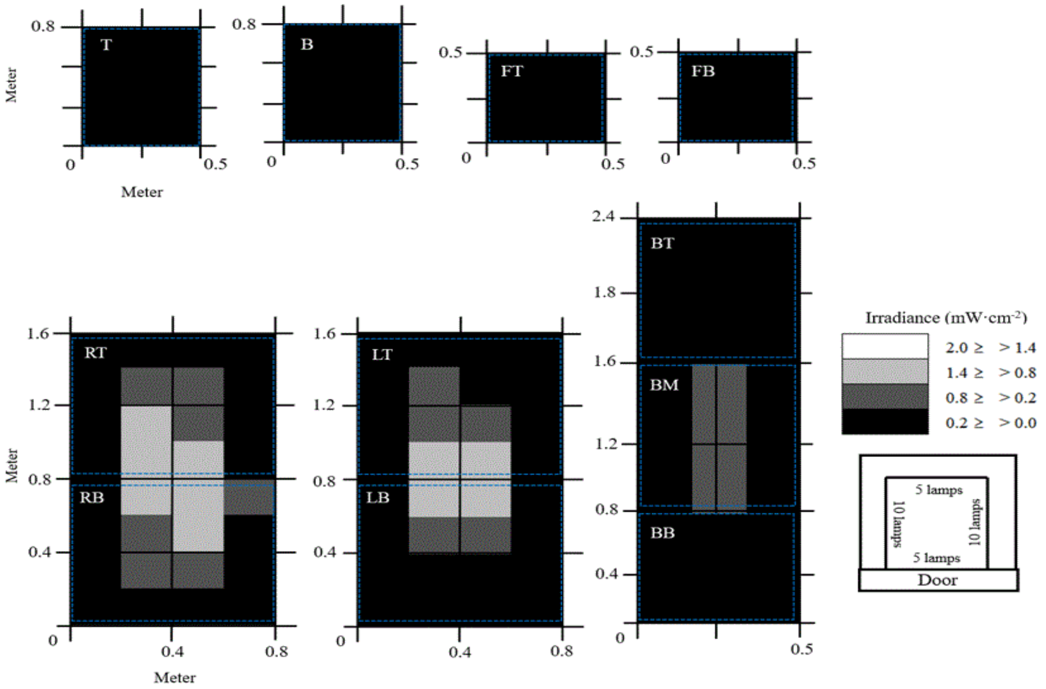

Figure S.9. The measured light intensity of photocatalysis. Map of UV-A light intensity measured on the surface of eleven panels inside a single chamber with 30 lamps.

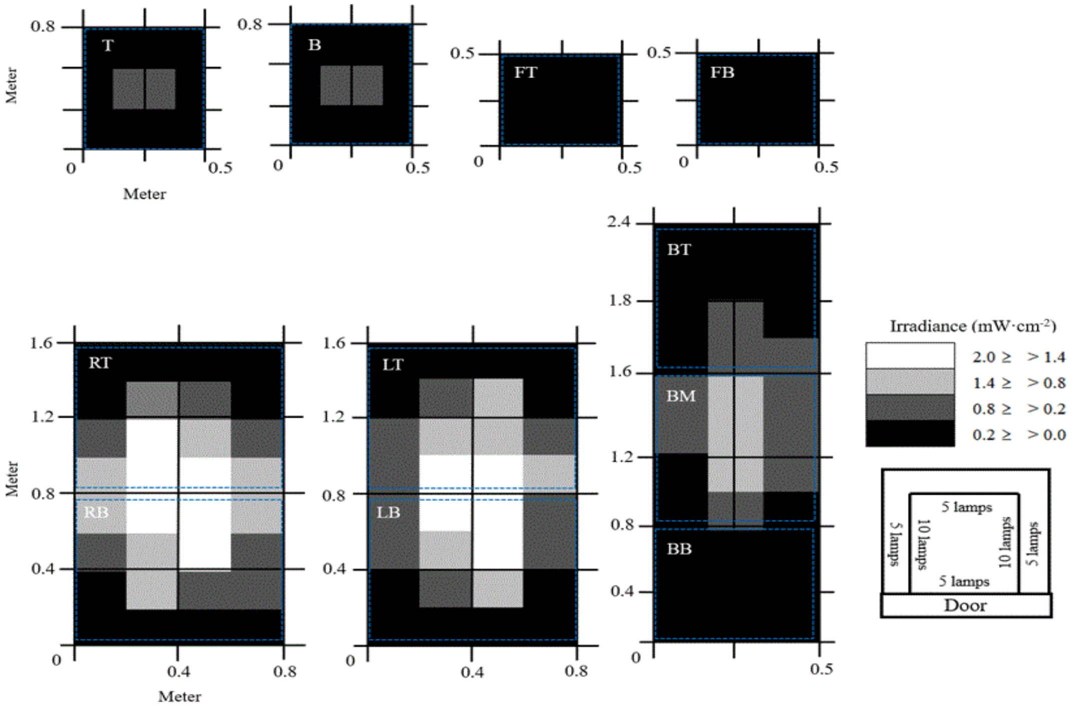

Figure S.10. The measured light intensity of photocatalysis. Map of UV-A light intensity measured on the surface of eleven panels inside a single chamber with 40 lamps.

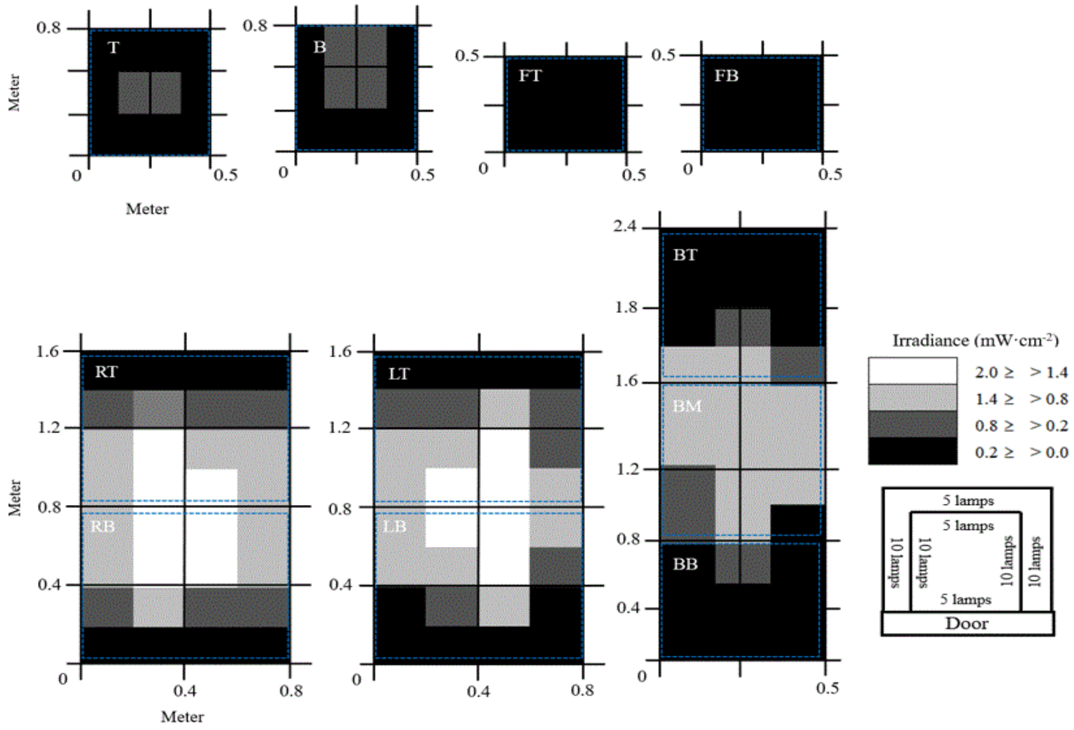

Figure S.11. The measured light intensity of photocatalysis. Map of UV-A light intensity measured on the surface of eleven panels inside a single chamber with 55 lamps.

Supplementary Material 6. Fan calibration

The system's volumetric flow rate was calibrated using the Fan Assessment Numeration System (FANS) unit, a portable fan tester [1-3]. The FANS unit incorporates a horizontal array of four propeller anemometers to create a real–time traverse of airflow entering the ventilation fan's 20-inch diameter (Figure S.13). The FANS unit consists of an open-ended box with smoothly curved inlet edges that are placed in front (intake side) of a fan [1]. This gives a velocity map across the face area that is used to calculate the volumetric flow rate entering the system. The measurable range of the FANS unit used is 500 to 10000 cubic feet per minute (CFM). Proof of sealed FANS unit attached to the system is provided (Figure S.12)

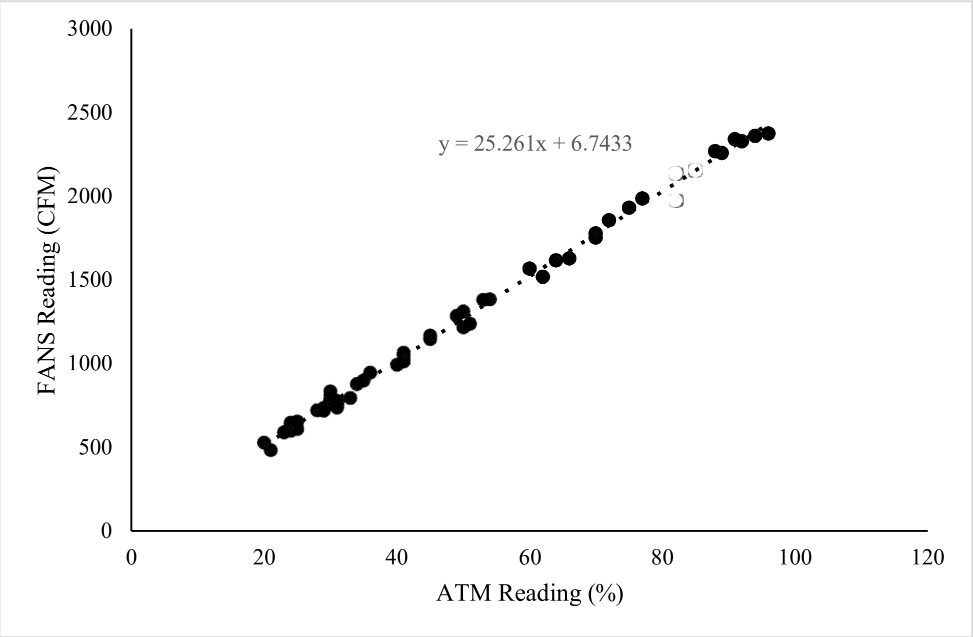

Figure S.12. Confirmation of the airflow control. Comparison of the external FANS unit measurement of airflow with the airflow control built in the system (ATM).

Prior to calibration, a leak test of our test chambers was performed. This was done to verify that all volumetric flow rate monitored by the FANS unit is entering the inlet and exiting the exhaust at the same rate. The leak test was performed by closing off the exhaust outlet, turning both fans to 100% operating speeds, and monitoring the ATM and FANS unit data. With an ATM reading of 0% of maximum volumetric flow and visual confirmation that there was no spin of the ATM fan blades, while the FANS unit recorded no incoming volumetric flow, the system was verified to have no leaks.
The process of the calibration was mostly automated using the FANS unit and system. The process began with the prop propellers at the top and slowly moves down through the FANS unit recording the openings air velocity data. The test is then run again at the same mobile lab fan setting only moving the props from the bottom of the unit to the top recording the same data. These two recorded airflows (CFM) are then compared to find the percent of error between the readings. If the error was above 5%, the procedure was done again to add to the amount of data being used for an average volumetric flow rate for the specific system setting. The 5% error was used to keep the hysteresis of the calibration as close as possible between upward and downward tests.
The FANS calibration flow rates were then added to a real-time recording percent of the ATM of the fan's maximum volumetric flow rate from the Lumina controller. By comparing the ATM percent of max flow rate vs. the FANS gold standard reading, a trendline could then be added to the data. Using the equation created by the trendline, the volumetric flow rate can be calculated based on the percent reading of the ATM that is displayed on the Lumina controller. Using this method to monitor the system volumetric flow rate allows the two fans at the inlet and exhaust of the system to operate at different variable speeds. This difference in fan speed allows the system to create a negative or positive pressured airflow ventilation. To creating the negative-pressure ventilation condition, the exhaust fan would be set to a higher speed than the inlet and vice versa to create pressure in the chambers. The method of creating the negative-pressure ventilation condition will be used to protect the user of the system against any harmful toxins created by treatment.

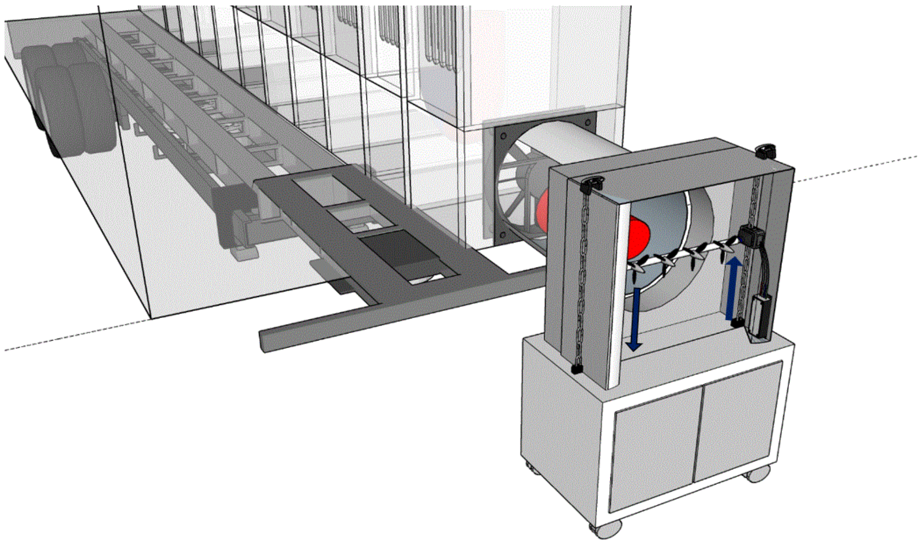

Figure S.13. Calibration of fan flow rate using a portable fan tester. The fan flow was measured using a fan airflow numeration system (FANS) that showed the volume metric flow rate.

The flow rate formed by adjusting two fans in the Lumina controller showed a very high correlation and high accuracy with the volumetric airflow measured by the FANS unit (Figure S.14). The error of both velocity measurements when the propeller goes down and up was below 5%, and the average value was used. The treated airflow in the mobile lab can be adjusted from ~0.25 $m^3 \cdot s^{-1}$ (535 CFM) to ~1.23 $m^3 \cdot s^{-1}$ (2600 CFM).

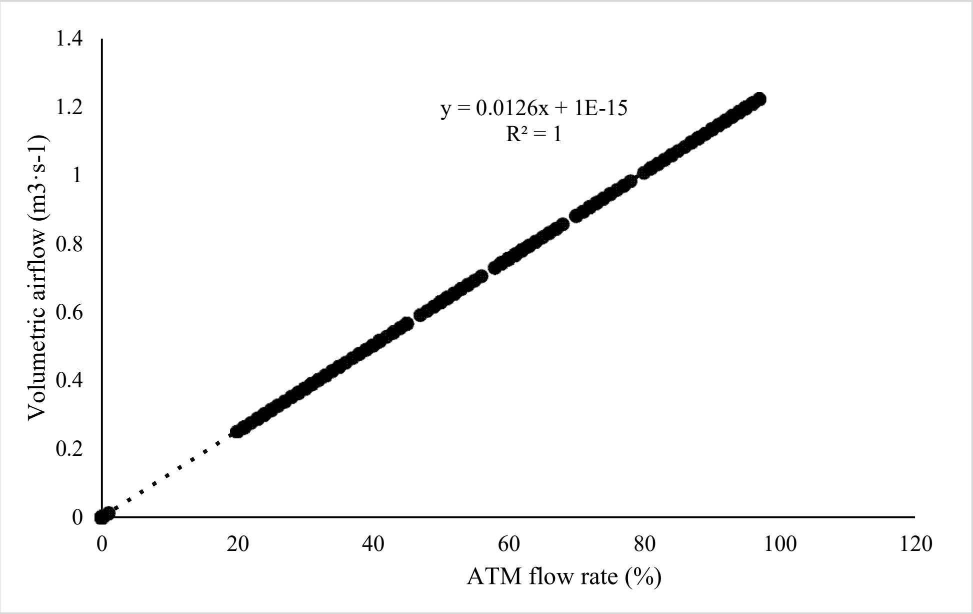

Figure S.14. Calibration of treated airflow through the UV mobile laboratory. Vertical axis = measured volumetric airflow with the FANS (fan airflow numeration system), Horizontal axis = % of flow rate (ATM %).

Supplementary Material 7. Experimental MERV filter setup

The mobile laboratory was designed and developed for the purpose of being used on a real farm site. However, the gas emitted by the exhaust fan of the actual farm contains various substances such as dust, manure powder, and flies. Therefore, it was considered that in order to accurately analyze UV treatment, it was necessary to remove various dust factors emitted from the farm. Therefore, in this study, a filtration unit was manufactured in a detachable form. Two types of Minimum Efficiency Reporting Value (MERV) filters were installed in the filtration unit (Figure S.15). Characteristics of the MERV filter type were summarized in Table S.9.

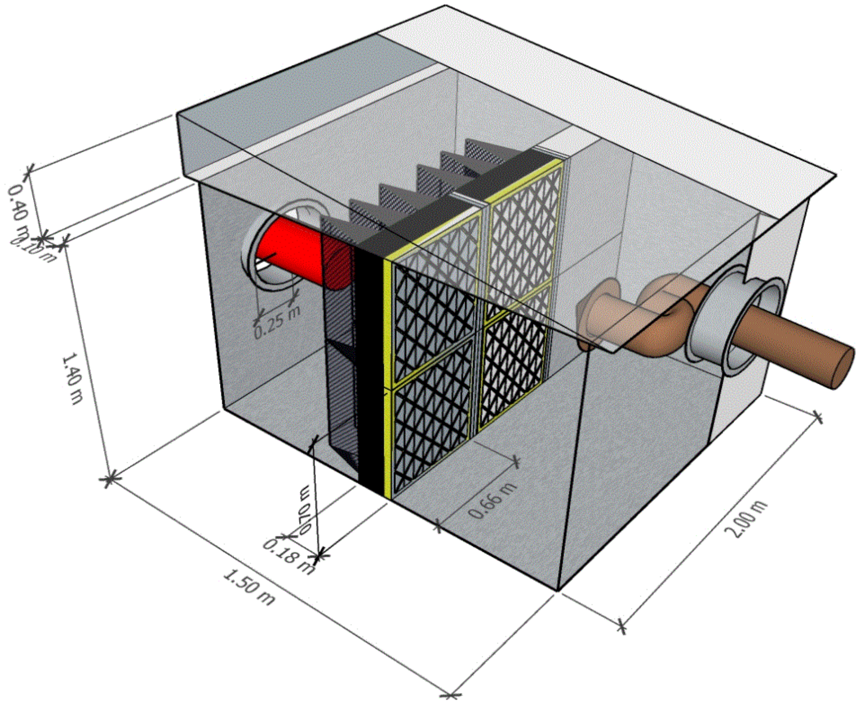

Figure S.15. Schematic of a flow-through filtration unit for trapping the airborne particulates (side view of filtration unit). A total of 8 minimum efficiency reporting value (MERV) filters (four #8 MERVs, yellow; and four #15 MERVs, black) were mounted inside the filtration unit. Treated air is filtered by #8 MERVs followed by #15 MERVs.

Table S.9. MERV filter rating.

MERV filter Ratin g	Average % of particle trapped efficiency (E) Particle matter (PM) size (μm) 0.3 - 1.0	1.0 - 3.0	3.0 - 10.0	Initial differential pressure (Pa)	Blocked substance
MERV 8	N/A	E < 20%	E > 70%	50	Lint, Dust, Mold spores
MERV 15	E < 85%	E > 90%	E >95%	140	Lint, Dust Mold spores, Smoke Bacteria, Virus Carriers

Supplementary Material 8. Evaluation of mobile UV laboratory's operation using standard gases.

The mobile laboratory and filtration unit are installed as in Figure S.16. When reflecting on the total volume of the mobile laboratory (14.4 m^3 without vertical baffles), connection (0.29 m^3) between the mobile lab and filtration unit, and filtration unit (4.0 m^3), the total treatment time was 74 seconds with the lowest airflow (0.25 $m^3 \cdot s^{-1}$), and the treatment time was 57 s in only the mobile laboratory under the lowest airflow (0.25 $m^3 \cdot s^{-1}$). Therefore, the treatment time per chamber was about 4.8 seconds. In order to check the operation of the mobile laboratory, untreated gas was introduced into the filtration unit. Untreated gas is NH_3 and Butan-1-ol standard gas as a control. The control was collected by measuring the concentration of the target gas in the mobile lab with the lamps turned off. Then, UV lamps were turned on, and the treated concentration was measured.

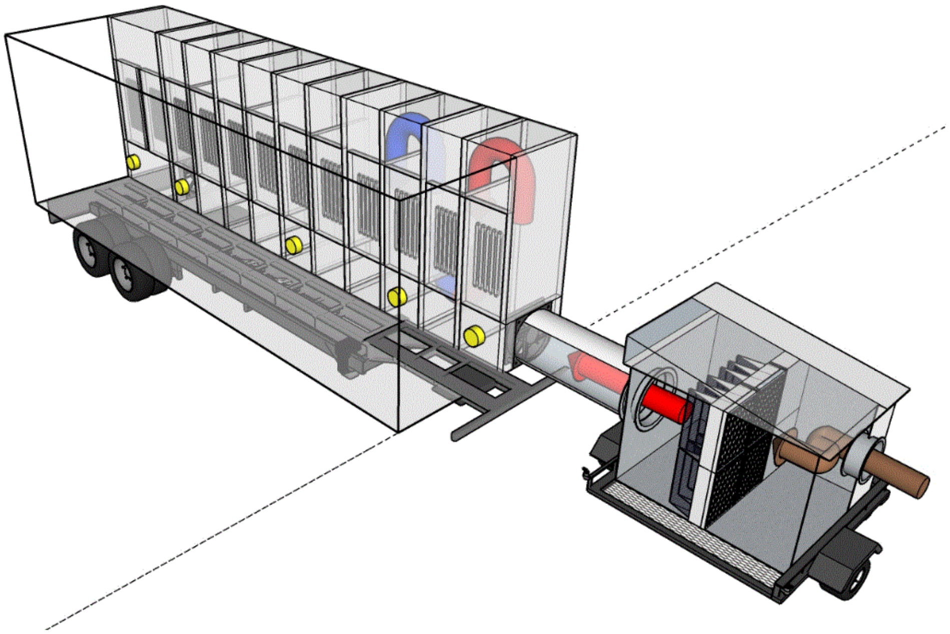

Figure S.16. Schematic of a flow-through UV mobile laboratory with a filtration unit. Brown arrow: inlet of untreated air; red arrow: inlet air with reduced particle matter load; blue arrow: treated air. The untreated air (brown arrow) could be either (a) standard gas, (b) mixture of standard gases, (c) surrogate odorous air, (d) exhaust from livestock barn, or other air pollution source. Yellow: air sampling port.

When NH_3 standard gas was injected into the filtration unit inlet, it took about 80 seconds to detect the equilibrated NH_3 concentration in a total of 12 chambers inside the mobile lab (Figure S.17).

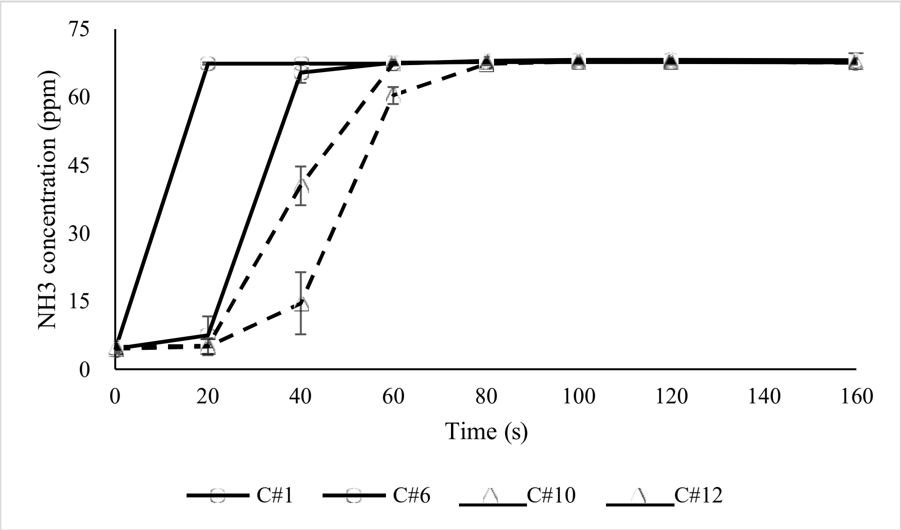

Figure S.17. Equilibration time of ammonia concentration in the mobile laboratory. Chamber #1 (chamber nearest to the air inlet), Chamber #6, Chamber #10, Chamber #12 (chamber nearest to the air outlet) signifies the location of air sampling ports. Airflow = 0.25 $m^3 \cdot s^{-1}$, temperature = 8 ℃, RH = 39%.

In the case of butan-1-ol, the concentration of the butan-1-ol sample was taken after the treatment time passed 80 seconds (Figure S.18). However, the concentration of butan-1-ol measured 80 seconds after injection of the standard gas showed a high standard deviation. The standard deviation decreased with increasing the equilibration time.

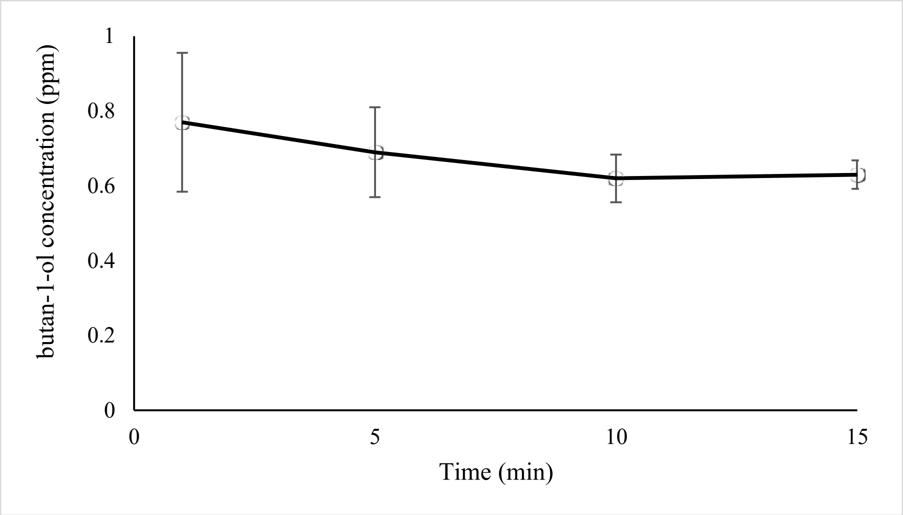

Figure S.18. Experimental determination of equilibration time of butan-1-ol standard gas concentration. Butan-1-ol was measured inside chamber #12 in the mobile laboratory to guide the experimental protocol for gas sampling. The variability of measured gas concentration and the concentration itself decreases with time. The results implied that gas sampling must be conducted after at least ~15 min delay to allow for the system to reach steady-state conditions. LED lamps off, Airflow = 0.25 $m^3 \cdot s^{-1}$, temperature = 11 ℃, RH =34%.

References

1. Gates, R.S.; Casey, K.D.; Xin, H.; Wheeler, E.F.; Simmons, J.D. Fan assessment numeration system (FANS) design and calibration specifications. *Transactions of the ASAE* **2004**, *47*, 1709.

2. Hoff, S.J.; Bundy, D.S.; Nelson, M.A.; Zelle, B.C.; Jacobson, L.D.; Heber, A.J.; Ni, J.; Zhang, Y.; Koziel, J.A.; Beasley, D.B. Real-time airflow rate measurements from mechanically ventilated animal buildings. *Journal of the Air & Waste Management Association* **2009**, *59*, 683-694.

3. Wheeler, E.F.; Gates, R.S.; Xin, H.; Zajaczkowski, J.S.; Topper, P.A.; Casey, K.D. Field estimation of ventilation capacity using FANS. In Proceedings of 2002 ASAE Annual Meeting; p. 1.
